# Proposals to Substitute an Elastic Cup Tor the Present Cupping–Glass

**Published:** 1853-11

**Authors:** Thomas Stratton

**Affiliations:** M.D. Edin.


From the London Lancet.

*Proposals to Substitute an Elastic Cup for the present Cupping-Glass.*

Much credit is due to the inventor of the "atmospheric breast-pump," made of a galvanized india-rubber globe, which, being tightly compressed, and its mouth-piece adapted to the nipple, resumes its form of a globe on the pressure being withdrawn; the milk then flowing into it to fill the vacuum.

Instead of the present plan of using the spirit-lamp or spirits of wine before applying the cupping-glass, would it not be much simpler and better to have cups made of the same substantial and elastic material, which answers so well when applied to the breast?

THOMAS STRATTON, M.D. Edin.,

